# Utilization and Spending With Preventive Drug Lists for Asthma Medications in High-Deductible Health Plans

**DOI:** 10.1001/jamanetworkopen.2023.31259

**Published:** 2023-08-29

**Authors:** Anna D. Sinaiko, Dennis Ross-Degnan, J. Frank Wharam, Robert F. LeCates, Ann Chen Wu, Fang Zhang, Alison A. Galbraith

**Affiliations:** 1 Department of Health Policy & Management, Harvard T.H. Chan School of Public Health, Boston, Massachusetts; 2 Department of Population Medicine, Harvard Medical School and Harvard Pilgrim Health Care Institute, Boston, Massachusetts; 3 Department of Medicine, Duke University, Durham, North Carolina; 4 Duke-Margolis Center for Health Policy, Durham, North Carolina; 5 Department of Pediatrics, Boston Medical Center and Boston University Chobanian & Avedisian School of Medicine, Boston, Massachusetts

## Abstract

**Importance:**

High-deductible health plans with health savings accounts (HDHP-HSAs) incentivize patients to use less health care, including necessary care. Preventive drug lists (PDLs) exempt high-value medications from the deductible, reducing out-of-pocket cost sharing; the associations of PDLs with health outcomes among patients with asthma is unknown.

**Objective:**

To evaluate the associations of a PDL for asthma medications on utilization, adverse outcomes, and patient spending for HDHP-HSA enrollees with asthma.

**Design, Setting, and Participants:**

This case-control study used matched groups of patients with asthma before and after an insurance design change using a national commercial health insurance claims data set from 2004-2017. Participants included patients aged 4 to 64 years enrolled for 1 year in an HDHP-HSA without a PDL in which asthma medications were subject to the deductible who then transitioned to an HDHP-HSA with a PDL that included asthma medications; these patients were compared with a matched weighted sample of patients with 2 years of continuous enrollment in an HDHP-HSA without a PDL. Models controlled for patient demographics and asthma severity and were stratified by neighborhood income. Analyses were conducted from October 2020 to June 2023.

**Exposures:**

Employer-mandated addition of a PDL that included asthma medications to an existing HDHP-HSA.

**Main Outcomes and Measures:**

Outcomes of interest were utilization of asthma medications on the PDL (controllers and albuterol), asthma exacerbations (oral steroid bursts and asthma-related emergency department use), and out-of-pocket spending (all and asthma-specific).

**Results:**

A total of 12 174 participants (mean [SD] age, 36.9 [16.9] years; 6848 [56.25%] female) were included in analyses. Compared with no PDL, PDLs were associated with increased rates of 30-day fills per enrollee for any controller medication (change, 0.10 [95% CI, 0.03 to 0.17] fills per enrollee; 12.9% increase) and for combination inhaled corticosteroid long-acting β2-agonist (ICS-LABA) medications (change, 0.06 [95% CI, 0.01 to 0.10] fills per enrollee; 25.4% increase), and increased proportion of days covered with ICS-LABA (6.0% [0.7% to 11.3%] of days; 15.6% increase). Gaining a PDL was associated with decreased out-of-pocket spending on asthma care (change, −$34 [95% CI, −$47 to −$21] per enrollee; 28.4% difference), but there was no significant change in asthma exacerbations and no difference in results by income.

**Conclusions and Relevance:**

In this case-control study, reducing cost-sharing for asthma medications through a PDL was associated with increased adherence to controller medications, notably ICS-LABA medications used by patients with more severe asthma, but was not associated with improved clinical outcomes. These findings suggest that PDLs are a potential strategy to improve access and affordability of asthma care for patients in HDHP-HSAs.

## Key Points

**Question:**

Is a preventive drug list (PDL) that exempts asthma medications from out-of-pocket cost sharing associated with utilization, adverse outcomes, and out-of-pocket spending for patients with asthma?

**Findings:**

In this case-control study including 12 174 participants with high-deductible health plans including health saving accounts, the PDL was associated with statistically significant increases in controller medication fills, increased adherence to combination inhaled corticosteroid long-acting β2-agonist medications, and decreased out-of-pocket spending on asthma care. There were no differences in asthma exacerbations.

**Meaning:**

This case-control study found that a PDL was associated with improved affordability of asthma medications and increased controller medication use but no difference in clinical asthma outcomes.

## Teaser

This case-control study evaluates whether inclusion of a preventive drug list for asthma medications is associated with utilization, adverse outcomes, and patient spending among patients with asthma enrolled in in high-deductible health plans with health savings accounts.

## Introduction

Asthma is a major cause of preventable disease burden with significant socioeconomic disparities.^1,2,3,4^ More than 25 million people in the US have asthma, including 8% of adults.^5^ Despite guidelines recommending use of controller medications, such as inhaled corticosteroids (ICS), leukotriene inhibitors (LTI), or combination ICS and long-acting β2-agonists (ICS-LABA) with demonstrated efficacy, adherence is suboptimal, increasing risk for asthma exacerbations, emergency department (ED) visits, hospitalizations, and death.^6,7,8,9,10,11,12,13^ High out-of-pocket (OOP) costs can be a barrier to obtaining needed medications for asthma and are associated with patients’ decreased medication use and adverse asthma outcomes.^14,15,16,17,18,19,20^

Over the past 20 years, commercial insurance designs have increased the share of costs paid by patients.^21^ Enrollees in high-deductible health plans (HDHP) that are eligible for a health savings account (HSA), with minimum deductibles of $1400 per individual in 2020 to 2022, are particularly at risk of cost-related barriers, because most medications are subject to the deductible. Controller medications are often expensive branded drugs without available generic substitutes.^22,23^ HDHP-HSAs are prevalent: in 2022, 25% of all firms offering health benefits offered one and 24% of covered workers were enrolled in these plans.^21^

Preventive drug lists (PDLs) are a form of value-based insurance design (VBID) and aim to preserve use of important medications through elimination of cost-related barriers to care.^24,25,26,27^ PDLs, which are lists of medications made exempt from the deductible in HDHP-HSAs and available at reduced or no cost,^28,29^ are increasingly popular.^27,30^ Evidence is mixed about the associations of reducing or eliminating cost-sharing with medication adherence and outcomes,^19,31,32,33,34,35,36^ and we lack evidence about the associations of PDLs in HDHP-HSAs with asthma outcomes. In this study, we analyze whether a PDL that includes asthma controller and rescue medications is associated with improved adherence to controller medications, reduced adverse health outcomes, and reduced patient OOP spending among commercially insured patients with asthma enrolled in these plans overall and for lower-income subgroups.

## Methods

This case-control study was approved with a waiver of informed consent by the Harvard Pilgrim Health Care institutional review board. Informed consent was waived because data were deidentified. This analysis followed the Strengthening the Reporting of Observational Studies in Epidemiology (STROBE) reporting guideline.

### Data and Study Groups

We used claims data set from 2004 to 2017 from a large US commercial and Medicare Advantage health plan with members in all 50 states, capturing enrollment and medical and pharmacy claims data. We included patients ages 4 to 64 years with at least 24 months continuous enrollment in an HDHP-HSA, during which they had at least 1 claim in the first 6 months for an outpatient visit, ED visit, or hospitalization with an asthma diagnosis (*International Classification of Diseases, Ninth Revision *[*ICD-9*] code 493.XX and *International Statistical Classification of Diseases and Related Health Problems, Tenth Revision *[*ICD-10*] code J45.XXX). We excluded enrollees with a diagnosis code for another serious comorbid pulmonary condition in the same enrollment period as their asthma diagnosis.^15,37,38^

We imputed whether the patient’s employer offered a PDL that included asthma medications based on deductible and copayment amounts on medication claims for all members within an employer account, based on methods in previously published work.^39^ Specifically, we identified in pharmacy claims all asthma medications included on the insurance carrier’s PDL using the National Drug Data File Plus (First DataBank). We measured deductibles and copayments paid for these asthma medications. Using rules based on the percentages of claims with deductibles and copayments for PDL-listed and unlisted medications (eMethods in Supplement 1),^39^ we identified periods in which eligible employers offered a PDL with asthma medications. Enrollees in HDHP-HSA plans with a PDL that did not include asthma medications were excluded. To reduce enrollee selection bias, we included only patients whose employers offered only HDHP-HSA plans in a study year, and did not offer a choice of plans with and without a PDL. Details on sample selection are provided in eFigure 1 in Supplement 1.

The PDL group included patients with a baseline period of 12 months in an HDHP-HSA without a PDL whose employers then added a PDL that included asthma controller and rescue medications for all members (ie, full replacement), after which they had continued enrollment for a follow-up period of 12 months (eFigure 2 in Supplement 1). The date of adoption of the PDL was considered the index date separating the 12-month baseline and follow-up periods. The control group was comprised of patients enrolled through employers in an HDHP-HSA plan without a PDL for 12 months before and after an employer anniversary month. If a member had multiple such 2-year blocks without a PDL, 1 block was randomly chosen. The plan anniversary date was considered the index date for control group enrollees.

We used the kmatch procedure in Stata version 16 (StataCorp) to balance employer- and patient-level characteristics between the study groups. Kmatch allows simultaneous exact matching and balancing on other variables included in a propensity score calculation. Variables included in the match were chosen based on theorized or demonstrated associations with HDHP-HSA enrollment or with outcomes.

We required sample members to match exactly on having an index date before or after October 2015 (transition from *ICD-9* to *ICD-10*), whether they were a child (age 4-17 years) or adult (age 18-64 years), time since first asthma diagnosis, use of each separate controller type (ICS, LTI, or ICS-LABA) interacted with month of first fill in the baseline period, baseline deductible level, follow-up period deductible level, and employer size (eMethods in Supplement 1).

We balanced the groups using entropy balanced weights emerging from the kmatch procedure, which results in equivalent weighted sample sizes and matching characteristics between PDL and control groups for all analyses.^40,41,42^ The propensity score used for entropy balancing included whether the patient had persistent asthma in the baseline period, patient index month, patient baseline annual OOP costs, an approximation of patient’s plan’s actuarial value (patient baseline OOP costs / patient’s standardized total costs), and plan type (eg, health maintenance organization, preferred provider organization). We identified individuals with persistent asthma in the baseline period using the Johns Hopkins ACG System (version 11.1)^43^ persistent asthma designation, which aligns with the Health Effectiveness Data and Information Set definition.^44,45,46^ The propensity score also included employer characteristics: mean employee age; percentage of employees by region, race and ethnicity, and census tract–level poverty; mean of employee baseline OOP costs and standardized total costs, and mean annual standardized costs per member per month. In subgroup analyses stratified by patients’ neighborhood income, we conducted separate matches within each income stratum using these methods.

### Outcomes

We had 4 categories of outcomes: controller medication fills and adherence, use of asthma rescue medications, asthma exacerbations, and OOP costs. Outcomes except OOP costs were measured in the baseline and follow-up periods, excluding the first and last month of each period to avoid stockpiling effects associated with the start or end of plan years in HDHPs.^39^ OOP costs were measured for the full 12 months of each period to capture total spending.

We measured counts of filled prescriptions for each asthma controller class (ICS, LTI, and ICS-LABA) and for rescue medications (albuterol and levalbuterol inhalers). For controllers, we used the days supplied for each fill to calculate the number of 30-day fills per patient per controller class in each study period. For rescue medications we measured number of fills per patient because these are acute medications for asthma exacerbations and are used as needed, not daily. We measured medication utilization outcomes for all patients, and among those with at least 1 controller medication fill during the baseline period.

We measured adherence using the proportion of days covered (PDC) for each controller class among enrollees with at least 1 fill in the first 6 months of the baseline period.^9,47^ Going forward from the first fill of the baseline period through the end of the follow-up period, we measured the days supplied for medications of that class in each study period. Because the number of days in the denominator varied based on the date of the first fill, we standardized the number of days supplied to a 365-day denominator, then calculated PDC as the proportion of days supplied out of 365.

Our proxy measures of asthma exacerbations included oral corticosteroid (OCS) bursts and asthma-related ED visits measured among those using controller medications at baseline. We defined OCS bursts as a dispensing of a 3- to 21-day supply after a nonuse period of at least 30 days without an OCS dispensing.^38^ We defined asthma-related ED visits as those with a principal diagnosis of asthma or with any diagnosis of asthma in combination with a diagnosis of a related condition (eMethods in Supplement 1).^37,38^

We measured OOP patient spending as the sum of deductible, copayment, and coinsurance amounts on claims separately in the baseline and follow-up periods. We measured enrollee OOP spending in total, for all asthma care (inpatient, outpatient, ED, medications, spacers, and nebulizers), for asthma medications (including controllers and rescue medications), for all controllers, for each controller type, and for rescue medications (eMethods in Supplement 1). Monthly spending within each spending measure was winsorized (a method of trimming to reduce the impact of outlier observations) to the 99th percentile of values greater than zero. Negative monthly spending values were set to zero. All OOP costs were adjusted to 2017 dollars using the medical consumer price index.^48^

### Covariates

In addition to the covariates used in matching, we measured other individual patient characteristics that were used to adjust the empirical models. Using 2008 to 2012 American Community Survey data at the census tract level we categorized enrollees’ neighborhood education according to the percentage of residents with less than a high school education and as low income if the percentage of households below the poverty level was at least 10.0%.^49,50^ Because race and ethnicity is a potential confounder of health care use, we used geocoding to classify participants as being from predominantly Black, Hispanic, White, or mixed race and ethnicity neighborhoods and used a superseding Hispanic or Asian categorization based on the E-Tech system (Ethnic Technologies), which analyzes full names and geographic locations of individuals, and recategorize them into being from predominantly Hispanic or Asian neighborhoods if indicated.^51,52,53,54^ Patient age and sex were extracted from enrollment files. We dropped patients with missing data on income, education, race and ethnicity, or region from the analyses (0.2% of the population).

### Statistical Analysis

We used a cohort study with matched groups to assess changes in outcomes from baseline to follow-up for patients in an HDHP-HSA who gained access to a PDL relative to controls who remained in an HDHP-HSA without PDL using a difference-in-differences design. Reported results are the mean association of the PDL intervention over our study period with study outcomes. A key assumption of this analytic method is that baseline (ie, preperiod) trends are parallel between the study groups.^55^ To test this assumption, we constructed interrupted time series with comparison series plots and differenced monthly PDL and control group outcomes; we used aggregate-level interrupted time series (ITS) analyses of the differenced trend to assess for parallel baseline trends.

We used generalized estimating equations, accounting for within-patient clustering over time applying the robust sandwich estimator and assuming an exchangeable working correlation structure.^56^ We used a negative binomial distribution for count outcomes (30-day fills, OCS bursts, and ED visits) and binomial distribution for PDC. The term of interest was the interaction between study group (PDL vs control) and time period (baseline vs follow-up year), and models were adjusted for individual patient characteristics not included in the match (ie, age, sex, race and ethnicity, income, education, region, index month and year, and Adjusted Clinical Group morbidity score). Using terms from the regression model, we applied marginal effects methods to calculate mean adjusted baseline and follow-up outcome rates and PDC as well as absolute and relative changes.^57^ We determined statistical significance using 2-sided *P* values adjusted for the Holm-Bonferroni correction for multiple comparisons^58^; 18 multivariable regression models were accounted for in the Holm-Bonferroni procedure. We stratified the analyses by whether patients lived in low-income neighborhoods. We did not conduct analyses stratified by age group (adults vs children) or analyses of PDC outcomes stratified by income level because of small subgroup samples sizes. Statistical analyses were conducted using Stata version 16. Data were analyzed from October 2020 to June 2023.

## Results

A total of 12 174 participants (mean [SD] age, 36.9 [16.9] years; 6848 [56.25%] female) were included in analyses. The weighted, matched study sample included 6087 enrollees in the PDL group and 6087 enrollees in the control group. On baseline individual patient characteristics not included in the match, groups were balanced as indicated by standardized mean differences less than 0.2 (Table 1).^59^ In the matched study sample, 2.44% of participants were Asian, 7.48% of participants were Hispanic, 6.99% of participants were non-Hispanic Black, and 78.3% of participants were non-Hispanic White; 85.7% of participants lived in neighborhoods with less than 15% of residents without a high school degree, and 29.3% of participants lived in low-income neighborhoods. One-third of participants (33.1%) had enrollment through small employers. ITS analyses demonstrated parallel baseline trends for all outcomes, with the exception of ICS-LABA controller fills, in which segmented regression analyses that adjusted for differing baseline trends were consistent with difference-in-differences analyses (eFigures 3-6 in Supplement 1).

**Table 1.** Baseline Characteristics of the Study Population Before and After Matching and Weighting

Characteristic	Before match			After matching and weighting			
	No. (%)		Standardized difference	No. (%)		Standardized difference
	PDL (n = 6477)	Control (n = 43 309)		PDL (n = 6087)	Control (n = 6087)	
Age at index date, y						
4-17	1088 (16.8)	12 113 (28.0)	0.215	987 (16.2)	934.7 (15.4)	0.047
18-29	1357 (21.0)	7313 (16.9)		1289 (21.2)	1430.3 (23.5)	
30-39	763 (11.8)	5637 (13.0)		729 (12.0)	822.9 (13.5)	
40-49	1251 (19.3)	7475 (17.3)		1174 (19.3)	1194.2 (19.6)	
50-64	2018 (31.2)	10 771 (24.9)		1908 (31.3)	1704.8 (28.0)	
Sex						
Male	2888 (44.6)	19 580 (45.2)	−0.029	2710 (44.5)	2616 (43.0)	−0.029
Female	3589 (55.4)	23 729 (54.8)		3377 (55.5)	3470.7 (57.0)	
Race and ethnicity						
Asian	150 (2.3)	1409 (3.3)	0.089	137 (2.2)	160.5 (2.6)	0.115
Hispanic	447 (6.9)	2662 (6.1)		422 (6.9)	488.6 (8.0)	
Non-Hispanic Black	395 (6.1)	2613 (6.0)		365 (6.0)	485.5 (8.0)	
Non-Hispanic White	5177 (79.9)	34 762 (80.3)		4873 (80.1)	4653.7 (76.5)	
Multiple	308 (4.8)	1863 (4.3)		290 (4.8)	298.6 (4.9)	
Neighborhood residents without high school degree, %^a^						
<15.0	5551 (85.9)	36 593 (85.7)	−0.004	5220 (86.0)	5161.2 (85.4)	−0.018
15.0-24.9	670 (10.4)	4632 (10.7)		625 (10.3)	637.3 (10.5)	
25.0-39.9	209 (3.2)	1313 (3.0)		200 (3.3)	217.3 (3.6)	
≥40.0	29 (0.4)	220 (0.5)		27 (0.4)	28.0 (0.5)	
Neighborhood residents below poverty line, %^a^						
<5.0	2006 (31.1)	13 844 (32.1)	0.017	1887 (31.1)	1660.7 (27.4)	−0.080
5.0-9.9	1950 (30.2)	12 854 (29.8)		1832 (30.2)	1901. 2 (31.4)	
10.0-19.9	1706 (26.4)	11 118 (25.8)		1607 (26.5)	1621.6 (26.8)	
≥20	797 (12.3)	5301 (12.3)		746 (12.3)	868.2 (14.3)	
Mean morbidity score (SD)	1.12 (1.93)	1.18 (2.02)	−0.034	1.08 (1.88)	1.11 (2.02)	−0.016
US region						
Midwest	1922 (29.7)	16675 (38.5)	−0.224	1800 (29.6)	1959.7 (32.2)	−0.118
Northeast	288 (4.4)	4209 (9.7)		256 (4.2)	544.4 (8.9)	
South	2678 (41.3)	15 325 (35.4)		2537 (41.7)	2234.7 (36.7)	
West	1589 (24.5)	7098 (16.4)		1494 (24.5)	1348.2 (22.2)	
Employer size, No. of employees						
1-99	2225 (34.4)	15 468 (35.7)	0.029	2014 (33.1)	2015.0 (33.1)	0.000
≥100	4252 (65.6)	27 841 (64.3)		4073 (66.9)	4072.0 (66.9)	

Abbreviation: PDL, preventive drug list.

^a^ Missing data: 209 observations on neighborhood-level education; and 210 observations on neighborhood-level poverty.

Patients who gained a PDL decreased annual OOP spending for their asthma care compared with patients in the control group (Table 2), including for all asthma medications combined (−$28 [95% CI, −$37 to −$18] per member; relative change, −32.4%), and all asthma care (−$34 [95% CI, −$47 to −$21] per member; relative change, −28.4%). OOP spending was also significantly lower for LTI medications (−$6 [95% CI, −$9 to −$3] per member; relative change, −36.9%) and for albuterol inhalers (−$8 [95% CI, −$10 to −$5] per member; relative change, −36.0%). There were no differences in total (ie, all medical and pharmacy care) OOP spending between groups.

**Table 2.** Adjusted Mean Out-of-Pocket Costs Before and After Adding a PDL to a High Deductible Health Plan With Health Savings Account Compared With a Control Group Remaining in a High Deductible Health Plan With Health Savings Account Without a PDL^a^

Medication or service	Mean out-of-pocket cost, $				Change for PDL vs control group	
	PDL		Control			
	Pre-PDL	Post-PDL	Before index date	After index date	Absolute, $ (95% CI)	Relative, % (95% CI)
**Full population** ^b^						
Controller medications						
ICS	14	9	15	13	−4 (−9 to 0)	−33.5 (−56.7 to −10.4)
LTI	20	10	15	12	−6 (−9 to −3)^c^	−36.9 (−50.9 to −23.0)^c^
ICS-LABA	32	22	31	29	−8 (−15 to −2)^c^	−27.8 (−44.4 to −11.1)^c^
Albuterol inhalers	20	14	21	22	−8 (−10 to −5)^c^	−36.0 (−44.1 to −27.9)^c^
All asthma medications	92	58	87	81	−28 (−37 to −18)^c^	−32.4 (−40.4 to −24.4)^c^
All asthma care	130	85	122	112	−34 (−47 to −21)^c^	−28.4 (−36.8 to −20.1)^c^
Total	1573	1538	1500	1541	−78 (−171 to 16)	−4.8 (−10.4 to 0.7)
**Among those with any controller use in baseline** ^d^						
Controller medications						
ICS	78	37	85	57	−15 (−34 to 4)	−29.3 (−57.0 to −1.7)
LTI	117	50	82	56	−30 (−46 to −14)^c^	−37.2 (−51.2 to −23.1)^c^
ICS-LABA	180	100	168	134	−44 (−76 to −12)^c^	−30.5 (−47.3 to −13.8)^c^
Albuterol inhalers	47	31	45	48	−20 (−28 to −11)^c^	−38.7 (−50.3 to −27.2)^c^
All asthma medications	432	225	391	303	−109 (−154 to −64)^c^	−32.7 (−42.5 to −22.8)^c^
All asthma care	532	302	473	377	−123 (−180 to −66)^c^	−29.0 (−39.2 to −18.8)^c^
Total	2190	1960	2182	2062	−110 (−283 to 63)	−5.3 (−13.3 to 2.7)

Abbreviations: ICS, inhaled corticosteroid; ICS-LABA, inhaled corticosteroid long-acting β agonist; LTI, leukotriene inhibitor; PDL, preferred drug list.

^a^ Sample sizes are matched and weighted.

^b^ Includes 6087 participants in each group.

^c^ Statistically significant after Holm-Bonferroni correction.

^d^ Includes 1098 participants in each group.

Among patients who were controller users at baseline, the PDL group had reductions in annual OOP spending on all asthma medications combined (−$109 [95% CI, −$154 to −$64] per member; relative change, −32.7%), all asthma care (−$123 [95% CI, −$180 to −$66] per member; relative change, −29.0%), LTI medications (−$30 [95% CI, −$46 to −$14] per member; relative change, −37.2%), and albuterol (−$20 [95% CI, −$28 to −$11] per member; relative change, −38.7%) compared with those in the control group (Table 2). Results were not substantively different among subgroups from low-income neighborhoods (eTables 1-3 in Supplement 1).

There were significant annual increases for the PDL group in 30-day fill rates for ICS-LABA (0.06 [95% CI, 0.01 to 0.10] fills per member; relative change, 25.4%) and for any controller (0.10 [95% CI, 0.03 to 0.17] fills per member; relative change, 12.9%) compared with controls (Table 3). Among controller users, 30-day fill rates increased for ICS-LABA (0.25 [95% CI, 06 to 0.44] fills per member; relative change, 24.6%) and for any controller (0.42 [95% CI, 0.07 to 0.78] fills per member; relative change, 11.6%) compared with baseline (Table 4). There was an increase in PDC for ICS-LABA (absolute change, 6.0% [95% CI, 0.7% to 11.3%]; relative change, 15.6%). There were no significant differences in 30-day fills or PDC for other controllers, albuterol fills, or rates of OCS bursts or asthma-related ED visits for the PDL group compared with controls.

**Table 3.** Adjusted Rates of Asthma Controller Medication Use Before and After Adding a PDL to a High Deductible Health Plan With Health Savings Account Compared With a Control Group Remaining in a High Deductible Health Plan With Health Savings Account Without a PDL^a^

Medication	30-d fills per enrollee, mean				Change for PDL vs control group	
	PDL		Control			
	Pre-PDL	Post-PDL	Before index date	After index date	Absolute mean rate per enrollee (95% CI)	Relative, % (95% CI)
Controller						
Any	0.78	0.86	0.75	0.73	0.10 (0.03 to 0.17)^b^	12.9 (2 to 24)^b^
ICS	0.14	0.15	0.13	0.13	0.00 (−0.02 to 0.03)	3.0 (−17.6 to 23.7)
LTI	0.42	0.44	0.40	0.39	0.04 (−0.01 to 0.09)	9.6 (−4.0 to 23.1)
ICS-LABA	0.22	0.27	0.22	0.21	0.06 (0.01 to 0.10)^b^	25.4 (2.3 to 48.6)^b^
Albuterol	0.50	0.49	0.52	0.51	0.00 (−0.05 to 0.05)	0.4 (−10.2 to 10.9)

Abbreviations: ICS, inhaled corticosteroid; ICS-LABA, inhaled corticosteroid long-acting β agonist; LTI, leukotriene inhibitor; PDL, preventive drug list.

^a^ Sample sizes are matched and weighted and include 6087 participants per group.

^b^ Statistically significant after Holm-Bonferroni correction.

**Table 4.** Adjusted Rates of Asthma Controller Medication Use, Adherence, and Exacerbations Before and After Adding a PDL to a High Deductible Health Plan With Health Savings Account Among Patients With Controller Medication Use in Pre-PDL Period Compared With a Control Group Remaining in a High Deductible Health Plan With Health Savings Account Without a PDL

Outcome	PDL			Control			Change for PDL vs control group	
	No.^a^	Pre-PDL	Post-PDL	No.^a^	Before index date	After index date	Absolute, estimate (95% CI)	Relative, % (95% CI)
30-d fills, mean rate per enrollee^b^								
Controller								
Any	1098	4.39	4.08	1099	4.10	3.40	0.42 (0.07 to 0.78)^c^	11.6 (1.2 to 22.1)^c^
ICS	1098	0.80	0.68	1099	0.72	0.57	0.05 (−0.09 to 0.18)	7.1 (−16.1 to 30.0)
LTI	1098	2.35	2.13	1099	2.20	1.88	0.13 (−0.11 to 0.37)	6.6 (−6.1 to 19.2)
ICS-LABA	1098	1.25	1.26	1099	1.17	0.95	0.25 (.06 to .44)^c^	24.6 (2.2 to 46.9)^c^
Albuterol	1098	1.28	1.21	1099	1.19	1.20	−0.07 (−0.25 to 0.11)	−5.6 (−19.1 to 7.9)
Days covered, %^d^								
ICS	135	51.5	37.0	140	44.7	29.1	3.4 (−5.2 to 12.1)	10 (−17.5 to 37.8)
LTI	389	70.3	58.4	392	72.7	59.1	1.3 (−4.4 to 7.0)	2.3 (−7.8 to 12.5)
ICS-LABA	234	55.9	44.3	237	58.6	40.1	6.0 (0.7 to 11.3)^c^	15.6 (0.3 to 30.9)^c^
Exacerbations^b^								
Any OCS bursts	1098	23.4	18.2	1099	22.1	16.3	0.9 (−4.7 to 6.5)	5.2 (−28.8 to 39.3)
Any asthma-related ED visits	1098	2.5	3.3	1099	3.5	4.5	0.1 (−2.6 to 2.8)	2.0 (−83.8 to 87.8)

Abbreviations: ED, emergency department; ICS, inhaled corticosteroid; ICS-LABA, inhaled corticosteroid long-acting beta agonist; LTI, leukotriene inhibitor; OCS, oral corticosteroid; PDL, preventive drug list.

^a^ Sample sizes are matched and weighted. PDL and control groups were matched separately for each controller subgroup for PDC analyses (eg, ICS users, LTI users, ICS-LABA users).

^b^ Among those with any controller fill in the baseline period.

^c^ Statistically significant after Holm-Bonferroni correction.

^d^ Among those with at least 1 fill in the first 6 months of the baseline (pre-PDL) period.

## Discussion

This case-control study found that exempting asthma medications from the deductible through PDLs was associated with a modest absolute increase in adherence to controller medications, particularly ICS-LABA, for which we found a 25% relative increase in fills. Access to the PDL also was associated with significantly reduced OOP spending on asthma care. There was no association of the PDL with use of asthma rescue medications or asthma exacerbations.

PDLs are a form of value-based insurance design that aim to remove cost-related barriers to efficacious preventive medications to improve access to care and outcomes for patients with chronic illness enrolled in HDHP-HSA plans. The positive association of PDLs with use of ICS-LABA medications is encouraging because this class of controller medications is recommended for patients with moderate or severe persistent asthma for its association with fewer severe exacerbations and symptom relief.^60^ It is also a more costly class of medications^22,61^ for which patients report affordability barriers.

The limited clinical outcomes associated with controller increases might have several causes. Most patients with asthma have mild disease and low levels of adherence to daily controller medication use,^9,62^ so the reduction in medication costs and modest increase in controller medication use in PDLs may not translate into a large difference in asthma outcomes. Targeting PDLs or other value-based insurance policies to patients most likely to experience cost-related nonadherence to asthma medications or those with clinical conditions that are more sensitive to cost-related nonadherence may increase the positive associations of these policies with clinical outcomes.

Asthma care may also be a priority for patients with more severe asthma who trade off other nonasthma care or other family needs to obtain needed asthma medications and care to avoid asthma flare-ups despite substantial financial strain.^63^ For these patients, the 28% reduction in OOP spending for asthma care may reduce other negative consequences of high health care costs, including struggling to pay for other important household expenses, such as food, housing, and utilities. Health plans with a PDL are more generous than those without, and employees may pay higher premiums to account for additional costs of these plans, which we cannot observe in our data. OOP asthma costs represent a more substantial portion of income for patients with lower income.^64,65^ However, the magnitudes of the observed outcomes were similar for patients in both high and low-income neighborhoods.

### Limitations

This study has some limitations. This is an observational study in which insurance plan type was not randomly assigned, and selection effects may exist. We mitigated this issue by including only employers that did not give enrollees a choice between the plan types of interest, and we observed only a single difference in preperiod trends between the PDL group and control group. However, we were unable to observe account balances in patients’ HSA accounts, and there is potential for differences across the study groups. For some less frequent outcomes, such as asthma-related ED visits, power to detect smaller differences was limited. We were unable to include hospitalizations as an outcome because of the small number of patients experiencing this outcome. With claims data, we were unable to measure certain outcomes, such as asthma control and missed work or school days. Like other studies using claims data, we are unable to determine the completeness of capture of all health care use by participants who have secondary coverage from another payer or paid out-of-pocket for care outside of their insurance coverage. We were also unable to measure whether patients in the PDL group were aware of the change in their benefit design, which has been noted in other studies and could have affected utilization of care.^66,67^ We lacked individual-level data on income and other socioeconomic characteristics but used well-established methods based on proxy census tract-level attributes.^38,49,54^ Findings may not be generalized to populations with public insurance, more severe asthma, more regular baseline controller use, or with comorbid pulmonary conditions.

## Conclusions

In this case-control study, adding a PDL to an HDHP-HSA plan was associated with increased adherence to asthma controller medications, particularly ICS-LABA used by patients with the moderate or severe persistent asthma, and significantly lower OOP spending for asthma care. There were no associations with utilization of other asthma medications or asthma exacerbations. Adopting VBID and other policies that exempt important medications for asthma medications are a potential strategy to reduce the cost-burden of asthma care and modestly improve adherence.

## Notes

Supplement 1.
eMethods.

eReferences.
**eFigure 1.** Cohort Selection **eFigure 2.** Study Insurance Design Change **eFigure 3.** Baseline Period Trends for Controller Medication Fills for PDL Group and Control Enrollees and the Difference Between Them **eFigure 4.** Baseline Period Trends for Albuterol Fills and Asthma Exacerbations for PDL Group and Control Enrollees and the Difference Between Them **eFigure 5.** Baseline Period Trends for Monthly OOP Costs for Controller Medications and Albuterol Inhalers for PDL Group and Control Enrollees and the Difference Between Them **eFigure 6.** Baseline Period Trends for OOP Costs for All Asthma Medications, All Asthma Care, and All Health Care Services for PDL Group and Control Enrollees and the Difference Between Them **eTable 1.** Subgroup Analyses Results: Asthma Controller Medication Use, Adherence, and Exacerbations Before and After Adding a PDL to an HSA-HDHP Compared With a Control Group Remaining in an HSA-HDHP Without a PDL, Low-Income Strata of Study Population **eTable 2.** Subgroup Analyses Results: Asthma Controller Medication Use, Adherence, and Exacerbations Before and After Adding a PDL to an HSA-HDHP Compared With a Control Group Remaining in an HSA-HDHP Without a PDL, High-Income Strata of Study Population **eTable 3.** Subgroup Analyses: Adjusted Mean Out-of-Pocket Costs Before and After Adding a PDL to an HSA-HDHP Compared With a Control Group Remaining in an HSA-HDHP Without a PDL

Supplement 2.
Data Sharing Statement
