# Supplementary material for: Utilization and Spending With Preventive Drug Lists for Asthma Medications in High-Deductible Health Plans
Source: JAMA Netw Open. 2023 Aug 29;6(8):e2331259. doi: 10.1001/jamanetworkopen.2023.31259 (PMC10466161; doi:10.1001/jamanetworkopen.2023.31259)
Supplement: Supplement 1. — eMethods. eReferences. eFigure 1. Cohort Selection eFigure 2. Study Insurance Design Change eFigure 3. Baseline Period Trends for Controller Medication Fills for PDL Group and Control Enrollees and the Difference Between Them eFigure 4. Baseline Period Trends for Albuterol Fills and Asthma Exacerbations for PDL Group and Control Enrollees and the Difference Between Them eFigure 5. Baseline Period Trends for Monthly OOP Costs for Controller Medications and Albuterol Inhalers for PDL Group and Control Enrollees and the Difference Between Them eFigure 6. Baseline Period Trends for OOP Costs for All Asthma Medications, All Asthma Care, and All Health Care Services for PDL Group and Control Enrollees and the Difference Between Them eTable 1. Subgroup Analyses Results: Asthma Controller Medication Use, Adherence, and Exacerbations Before and After Adding a PDL to an HSA-HDHP Compared With a Control Group Remaining in an HSA-HDHP Without a PDL, Low-Income Strata of Study Population eTable 2. Subgroup Analyses Results: Asthma Controller Medication Use, Adherence, and Exacerbations Before and After Adding a PDL to an HSA-HDHP Compared With a Control Group Remaining in an HSA-HDHP Without a PDL, High-Income Strata of Study Population eTable 3. Subgroup Analyses: Adjusted Mean Out-of-Pocket Costs Before and After Adding a PDL to an HSA-HDHP Compared With a Control Group Remaining in an HSA-HDHP Without a PDL [file jamanetwopen-e2331259-s001.pdf]

Supplemental Online Content

Sinaiko AD, Ross-Degnan D, Wharam JF, et al. Utilization and spending with preventive drug lists for asthma medications in high-deductible health plans. *JAMA Netw Open*. 2023;6(8):e2331259. doi:10.1001/jamanetworkopen.2023.31259

eMethods.
eReferences.
eFigure 1. Cohort Selection
eFigure 2. Study Insurance Design Change
eFigure 3. Baseline Period Trends for Controller Medication Fills for PDL Group and Control Enrollees and the Difference Between Them
eFigure 4. Baseline Period Trends for Albuterol Fills and Asthma Exacerbations for PDL Group and Control Enrollees and the Difference Between Them
eFigure 5. Baseline Period Trends for Monthly OOP Costs for Controller Medications and Albuterol Inhalers for PDL Group and Control Enrollees and the Difference Between Them
eFigure 6. Baseline Period Trends for OOP Costs for All Asthma Medications, All Asthma Care, and All Health Care Services for PDL Group and Control Enrollees and the Difference Between Them
eTable 1. Subgroup Analyses Results: Asthma Controller Medication Use, Adherence, and Exacerbations Before and After Adding a PDL to an HSA-HDHP Compared With a Control Group Remaining in an HSA-HDHP Without a PDL, Low-Income Strata of Study Population
eTable 2. Subgroup Analyses Results: Asthma Controller Medication Use, Adherence, and Exacerbations Before and After Adding a PDL to an HSA-HDHP Compared With a Control Group Remaining in an HSA-HDHP Without a PDL, High-Income Strata of Study Population
eTable 3. Subgroup Analyses: Adjusted Mean Out-of-Pocket Costs Before and After Adding a PDL to an HSA-HDHP Compared With a Control Group Remaining in an HSA-HDHP Without a PDL

This supplemental material has been provided by the authors to give readers additional information about their work.

© 2023 Sinaiko AD et al. *JAMA Network Open.*

eMethods

Imputation of Preventive Drug Lists (PDLs)

The 2004 Internal Revenue Service (IRS) regulations for Health Savings Accounts (HSAs) excluded pre-deductible coverage of medications to treat existing illnesses but did not provide a list of allowed medications. Some health plans, including the one in our study, included medications for secondary prevention of asthma and other chronic conditions on their PDLs. Of note, in 2019, the IRS issued guidance to permit HSA-eligible High-Deductible Health Plans (HSA-HDHPs) to include pre-deductible coverage of a list of medications to prevent exacerbations of chronic conditions, including inhaled corticosteroids for asthma.[1–3]

Because our data did not contain a benefits variable indicating whether a PDL was offered as part of an HSA-high-deductible plan, we used pharmacy claims to impute whether an employer offered a PDL, based on methods in the study team's previously published work.[2] Because larger employers are more likely to offer an extended pharmacy benefit that includes a PDL and are more likely to have enough pharmacy claims for reliable imputation, we restricted inclusion to employers with at least 100 enrollees (employees and family members) per month.

We required that eligible members in HSA-HDHP plans have no choice between plans with and without a PDL. We determined whether members had choice between plans by examining the number of members transitioning between contracts, especially during annual open enrollment. The national insurer represented in our data offered two standard types of PDLs: a core PDL and an expanded PDL. The core PDL contained medications in several therapeutic categories (i.e., for hypertension, high cholesterol, breast cancer prevention, blood clotting/platelet therapy, organ rejection, osteoporosis, prenatal vitamins) that employers

© 2023 Sinaiko AD et al. *JAMA Network Open.*

could choose to offer as a pharmacy benefit enhancement; the expanded PDL contained additional therapeutic categories (i.e., for diabetes, asthma, HIV/AIDS, multiple sclerosis, psychosis) that could be optionally added to the core list. Although the lists were standardized, employers had some discretion over the composition of the PDLs they offered. We had access to the insurer's annual core and expanded PDL lists for the years 2008-2014 and varied the products included accordingly; we used the 2008 list for earlier years. For our imputation, we included all products (brand or generic) that were chemically equivalent to medications listed on the core or expanded PDL using equivalency data from the First DataBank National Drug Data File PlusTM (First DataBank, Inc., San Bruno, CA).

In HSA-HDHPs, medications would normally be subject to the deductible, although they could be subject to co-payments after the deductible is met. Employers offering HSA-HDHPs linked to PDLs could choose different copayment arrangements for covered products but shared the common policy that medications on the PDL were exempt from deductibles throughout the benefit year. Since the percentage of members satisfying annual deductibles increases during the benefit year, it is harder later in the benefit year to determine whether no deductible payment on a claim means that the medication is covered under a PDL or that the annual deductible obligation has been met. We thus used only the first 6 months of the benefit year to separately impute the presence of a core or an expanded PDL. To be counted as a possible PDL month, we required that ≤10% of fills in the month for listed medications be charged a deductible and that >10% of claims for unlisted medications have a deductible charge. To be counted as a PDL year, at least 5 of the first 6 months in the year (or 4/5 if one month had no pharmacy claims) had to meet the monthly rule, the benefit year had to contain

© 2023 Sinaiko AD et al. *JAMA Network Open.*

a full 12 months, and <10% of total pharmacy claims for PDL listed medications during the entire year could have a deductible charge. Benefit years with a full 12 months of coverage in which deductibles were charged for >10% of listed medications were identified as non-PDL years.

We used the imputed annual PDL status to identify situations in which an employer had fully replaced a full year of coverage in an HSA-HDHP plan without a PDL with a full year of coverage in an HSA-HDHP plan with a PDL including asthma medications (i.e., exempting asthma medication from deductibles); the first day of the month of the coverage switch was defined as the index date. Using a common industry definition, full replacement was defined as at least 85% of enrollees in all contracts linked to an employer in the prior month being switched together to the new coverage. We also identified all situations in which employers had continued all employees in HSA-HDHP coverage without a PDL for a two-year period; if employers had more than one such two-year period, one was randomly selected to be included in the control pool, with the first day of the second year of coverage defined as the index date.

Methods used in Matching

We used a combination of exact and propensity matching methods to match the control group 1:1 to the PDL group. We additionally weighted the matched groups using entropy balanced weights emerging from the Stata kmatch matching procedure, which weight some control group patients more heavily in the analysis and results in equivalent weighted sample sizes between PDL and control groups for all analyses.[4,5] We chose variables for inclusion in the exact and propensity match based on prior studies using these matching methods for PDL analyses.[6] We chose variables for the exact match based on their actual or hypothesized strong

© 2023 Sinaiko AD et al. *JAMA Network Open.*

relationship with PDL enrollment, utilization outcomes, or baseline imbalance. Other potential confounders not used in the exact match were used for propensity score model.

Specifically we required sample members to match exactly on index date before or after October 2015 (transition from ICD-9 diagnosis codes to ICD-10 diagnosis codes), age group (child 4-17, adult 18-64), time of first asthma diagnosis (7-12 months prior to index date vs. 13+ months), use of each separate controller type (ICS, LTI, ICS-LABA) interacted with month of first fill in the baseline period, baseline deductible level ($0-500, $1000-2499, $2500+), follow-up deductible level, and employer size (1-100 vs. >100 employees). We directly measured deductible levels when available and otherwise imputed this information using aggregated out-of-pocket spending among enrollees within an employer.[7,8]

We also included the following enrollee characteristics in logistic regression models to create a propensity score that kmatch used for entropy balancing: whether the enrollee had persistent asthma in the baseline period, enrollee index month, enrollee's measured plan actuarial value (baseline out-of-pocket costs/standardized total costs), in categories (≤0.25, >0.25 to 0.66, >0.66), total annual out-of-pocket costs (<$500, $500-≤$1500, >$1500), baseline and follow-up plan type (Health Maintenance Organization (HMO), Preferred Provider Organization (PPO), etc.). Employer characteristics that contributed to the propensity score used for entropy balancing were as follows: mean employee age, percent of employees by region, race/ethnicity, and census tract poverty, ratio of out-of-pocket to standardized total cost for the employer for the entire baseline year (≤0.19, >0.19 to 0.28, >0.28) (Consumer Price Index (CPI) adjusted using June 2017 Medical CPI), mean annual employer standardized costs per member per month (< $350, $350 - <500, ≥ $500) (already CPI-adjusted). Variable cut-

© 2023 Sinaiko AD et al. *JAMA Network Open.*

points for categorical variables were chosen based on standard values (employer size, deductible levels) or to create relatively evenly distributed subgroups (e,g, OOP/standardized costs ratio). We used the overall matched population for the main analyses and rematched with low-income enrollees for sub-group analyses.

We used multivariate-distance kernel matching (kmatch, STATA) with entropy balancing at the second moment. The default weight one was given to the propensity score when computing distances. The resulting bandwidth was 8.689298*.* The Stata codes are shown below*:*

```
kmatch md $tx_var $subgroup_vars $exact_vars $match_vars, ematch($subgroup_vars
$exact_vars) ebalance targets(2) att wgen(_full_sample_wt) replace psvars($subgroup_vars
$exact_vars $match_vars) comsup
```

Defining service categories for asthma out-of-pocket spending

Patient out-of-pocket spending was reported directly for each medical and pharmacy claim in three fields: deductible spending, copayment spending, and coinsurance spending. We measured out-of-pocket spending as the sum of deductible, copayments, and coinsurance amounts on claims separately in the baseline and follow-up period using the full 12 months of data in each period to estimate annual spending. We measured enrollee out-of-pocket spending in total, and for all asthma care (inpatient, outpatient, Emergency Department (ED), medications), defined as follows:

Asthma-related inpatient services were defined as a hospitalization with a principal diagnosis of asthma; OR a diagnosis of asthma in any position AND a diagnosis of one of the following related conditions: acute respiratory infections (ICD-9 codes 460-466.xx)**,** chronic obstructive pulmonary disease and allied Conditions (ICD-9 codes 490-496.xx)**,** other conditions originating in the perinatal period (ICD-9 codes 764-779.xx)**,** other diseases due to viruses and

© 2023 Sinaiko AD et al. *JAMA Network Open.*

chlamydiae (ICD-9 codes 070-079.xx), other diseases of respiratory system (ICD-9 codes 510-519.xx), pneumonia and influenza (ICD-9 codes 480-488.xx).

Asthma-related outpatient visits were defined as office visits with a principal diagnosis of asthma.

Asthma-related emergency department (ED) visits were defined as an ED visit with a principal diagnosis of asthma; OR a diagnosis of asthma in any position AND a diagnosis of one of the following related conditions: acute respiratory infections (ICD-9 codes 460-466.xx), chronic obstructive pulmonary disease and allied Conditions (ICD-9 codes 490-496.xx), other conditions originating in the perinatal period (ICD-9 codes 764-779.xx), other diseases due to viruses and chlamydiae (ICD-9 codes 070-079.xx), other diseases of respiratory system (ICD-9 codes 510-519.xx), pneumonia and influenza (ICD-9 codes 480-488.xx).

Asthma-related medications included prescriptions filled for biologics (anti-IgE), controller medications (including inhaled corticosteroids (ICS), ICS long-acting beta agonists (ICS-LABA), long-acting beta agonists (LABA), leukotriene inhibitors (LTI), mast cell stabilizers, and xanthine), oral steroids, and rescue medications (including ipratropium, short-acting beta agonists (SABA), and ipratropium short-acting beta agonists (SABA ipratropium)).

Asthma-related durable medical equipment included prescriptions filled or medical claims for spacers for asthma inhalers and nebulizer machines.

Difference-in-Differences Analysis

We used a difference-in-differences design to assess changes in outcomes from baseline to follow-up for patients in an HSA-HDHP who gained access to a PDL relative to controls who remained in an HSA-HDHP without PDL. We estimated:

© 2023 Sinaiko AD et al. *JAMA Network Open.*

$$Y_{it} = \alpha_i + \beta_1 PDL_i + \beta_2 FollowUp_t + \beta_3 (PDL * FollowUp)_{it} + \boldsymbol{X\gamma} + \varepsilon_{it}$$

Where Y_{it} are study outcomes for person *i* in period t. $\boldsymbol{X\gamma}$ is a vector of covariates not included in the match and included age, sex, race/ethnicity, income, education, region, index month and year, and ACG morbidity score.

© 2023 Sinaiko AD et al. *JAMA Network Open.*

eReferences

1. Tanner S. *Part III - Administrative, Procedural, and Miscellaneous*. Office of Division Counsel/Associate Chief Counsel Accessed July 15, 2023. https://www.irs.gov/pub/irs-drop/n-04-23.pdf

2. Fischer W. *Additional Preventive Care Benefits Permitted to Be Provided by a High Deductible Health Plan Under § 223*. Office of Associate Chief Counsel; 2019. Accessed July 15, 2023. https://www.irs.gov/pub/irs-drop/n-19-45.pdf

3. Fronstin P, Roebuck MC, Fendrick AM. *Premium Impact of Expanding Pre-Deductible Coverage to Chronic Disease Management Medications in HSA-Eligible Health Plans*. EBRI Issue Brief; 2022.

4. Jann B. KMATCH: Stata Module Module for Multivariate-Distance and Propensity-Score Matching, Including Entropy Balancing, Inverse Probability Weighting, (Coarsened) Exact Matching, and Regression Adjustment. *Boston College Department of Economics*. Published online 2020.

5. Hainmueller J. Entropy Balancing for Causal Effects: A Multivariate Reweighting Method to Produce Balanced Samples in Observational Studies. *Polit Anal*. 2012;20(1):25-46.

6. Ross-Degnan D, Wallace J, Zhang F, Soumerai SB, Garabedian L, Wharam JF. Reduced Cost-sharing for Preventive Drugs Preferentially Benefits Low-income Patients With Diabetes in High Deductible Health Plans With Health Savings Accounts. *Medical Care*. 2020;58:S4-S13. doi:10.1097/MLR.0000000000001295

7. Wharam JF, Zhang F, Eggleston EM, Lu CY, Soumerai S, Ross-Degnan D. Diabetes outpatient care and acute complications before and after high-deductible insurance enrollment: A natural experiment for translation in diabetes (NEXT-D) study. *JAMA Internal Medicine*. 2017;177(3):358-368. doi:10.1001/jamainternmed.2016.8411

8. Wharam JF, Zhang F, Wallace J, et al. Vulnerable And Less Vulnerable Women In High-Deductible Health Plans Experienced Delayed Breast Cancer Care. 2020;38(3):408-415. doi:10.1377/hlthaff.2018.05026.Vulnerable

© 2023 Sinaiko AD et al. *JAMA Network Open.*

eFigure 1. Cohort selection

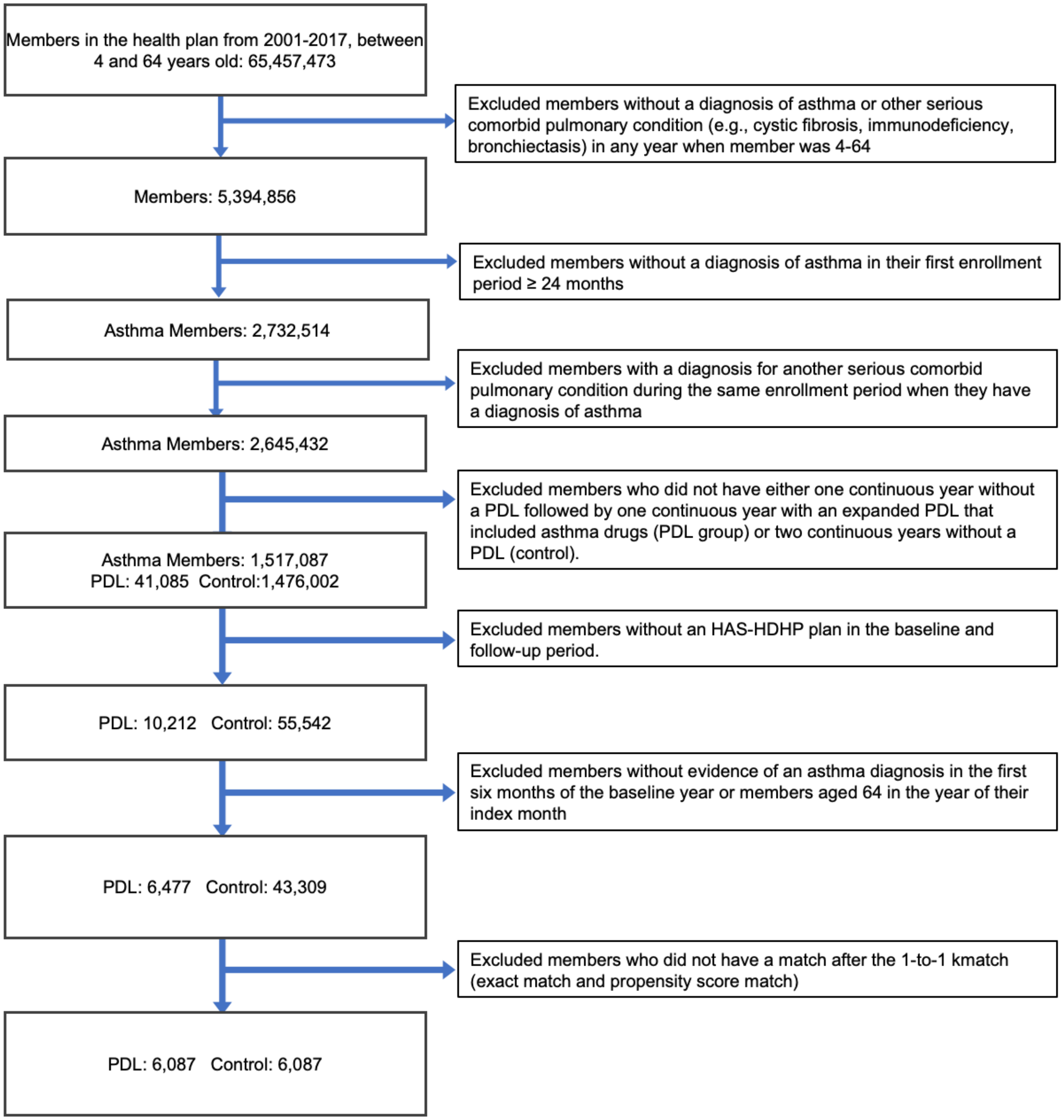

© 2023 Sinaiko AD et al. *JAMA Network Open.*

eFigure 2. Study Insurance Design Change

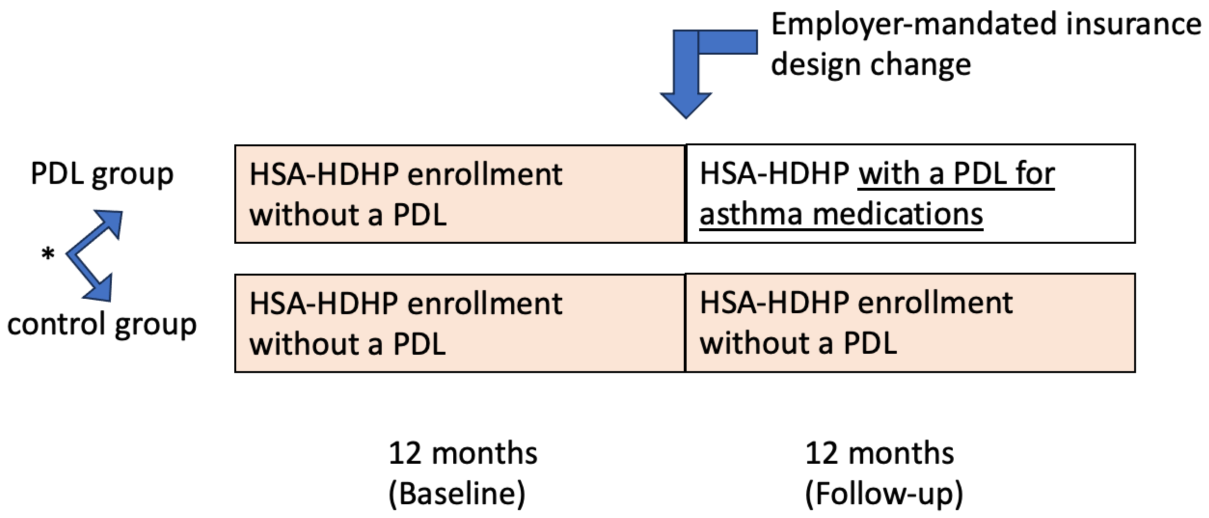

*STATA kmatch procedure applied, with exact match on index date before or after transition from ICD-9 to ICD-10, whether child (age 4-17) or adult (age 18-64), time since first asthma diagnosis, use of each separate controller type (ICS, LTI, ICS-LABA) interacted with month of first fill in the baseline period, deductible levels, and employer size; propensity score for entropy balancing included the following variables: whether the patient had persistent asthma in the baseline period, patient index month, patient baseline annual out-of-pocket costs, patient baseline out-of-pocket costs/patient's standardized total costs, and plan type, and employer characteristics (mean employee age, percent of employees by region, race/ethnicity, and census tract poverty, mean of employee baseline out-of-pocket costs/standardized total costs, and mean annual standardized costs per member per month.

PDL = Preferred Drug List, HSA-HDHP = High Deductible Health Plan with a Health Savings Account

© 2023 Sinaiko AD et al. *JAMA Network Open.*

eFigure 3. Baseline period trends for controller medication fills for PDL group and control enrollees and the difference between them.

ICS 30-day fills

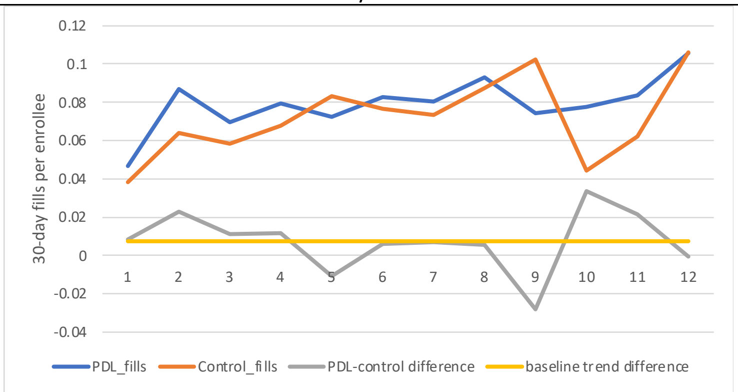

LTI 30-day fills

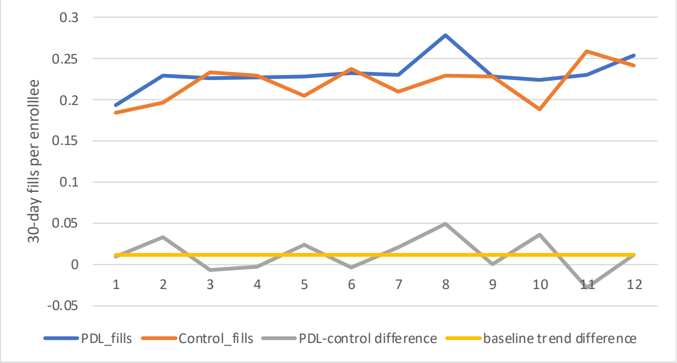

ICS-LABA 30-day fills

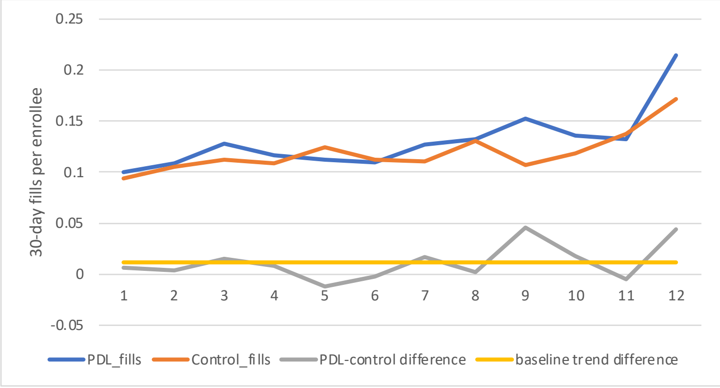

Abbreviations: ICS = inhaled corticosteroid; ICS-LABA = inhaled corticosteroid-long-acting beta agonist; LTI = leukotriene inhibitor; PDL = preventive drug list

© 2023 Sinaiko AD et al. *JAMA Network Open.*

eFigure 4. Baseline period trends for albuterol fills and asthma exacerbations for PDL group and control enrollees and the difference between them.

Albuterol Fills	Any OCS bursts
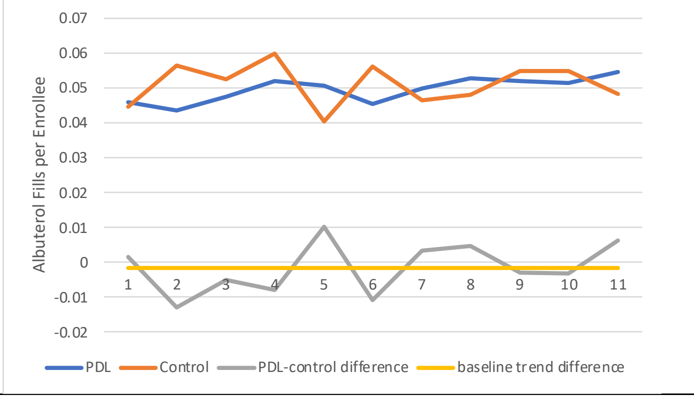	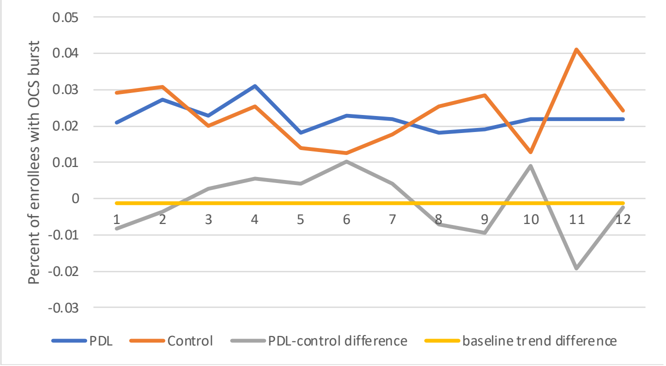
Asthma-related ED visits	
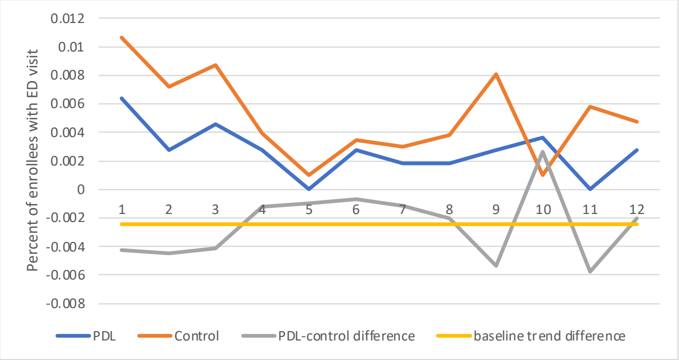	

Abbreviations: ED = emergency department; OCS = oral corticosteroid; PDL = preventive drug list

© 2023 Sinaiko AD et al. *JAMA Network Open.*

eFigure 5. Baseline period trends for monthly OOP costs for controller medications and albuterol inhalers for PDL group and control enrollees and the difference between them.

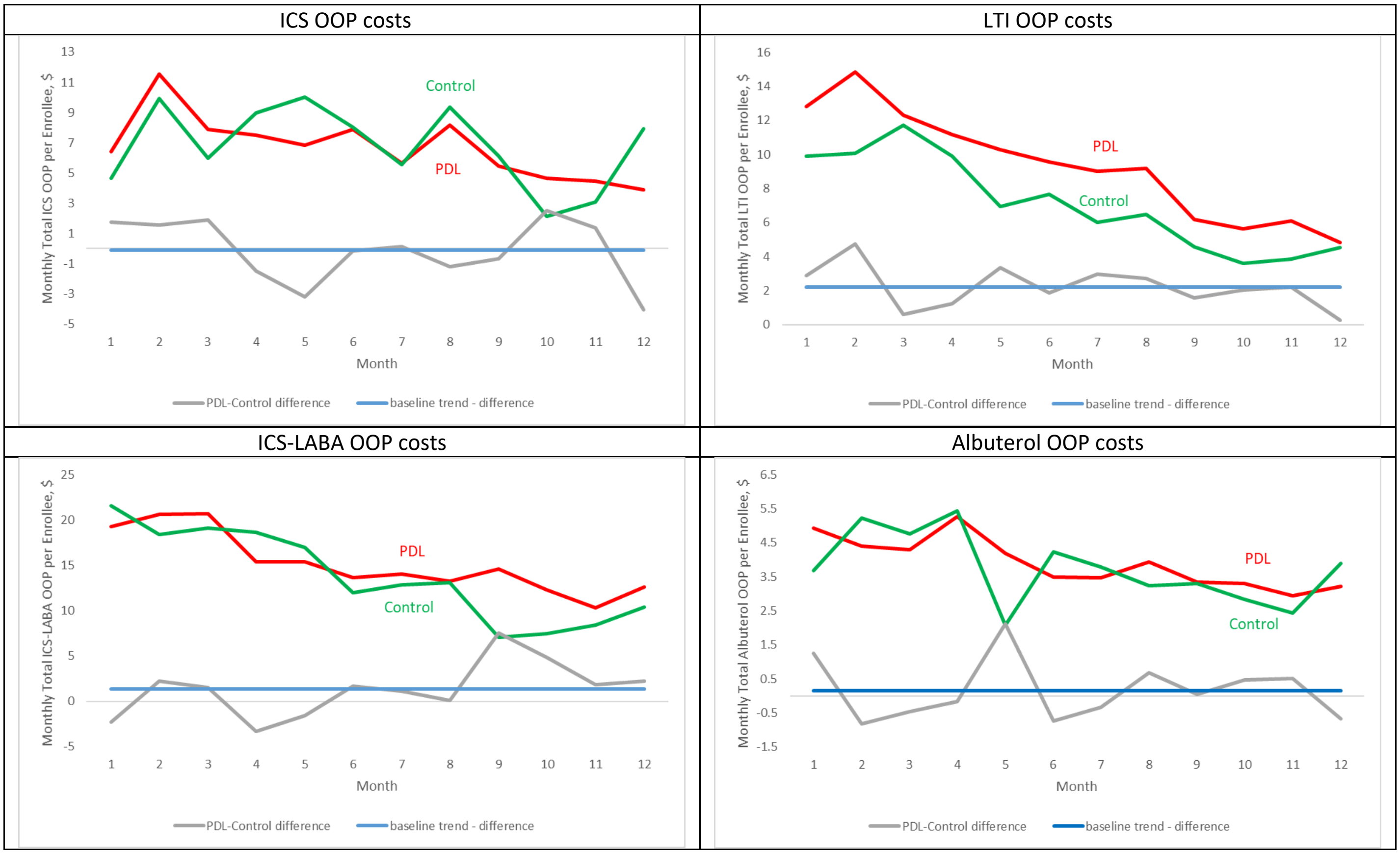

Abbreviations: ICS = inhaled corticosteroid; ICS-LABA = inhaled corticosteroid-long-acting beta agonist; LTI = leukotriene inhibitor; OOP = out-of-pocket; PDL = preventive drug list

© 2023 Sinaiko AD et al. *JAMA Network Open.*

eFigure 6. Baseline period trends for OOP costs for all asthma medications, all asthma care, and all health care services for PDL group and control enrollees and the difference between them.

Total asthma pharmacy OOP costs

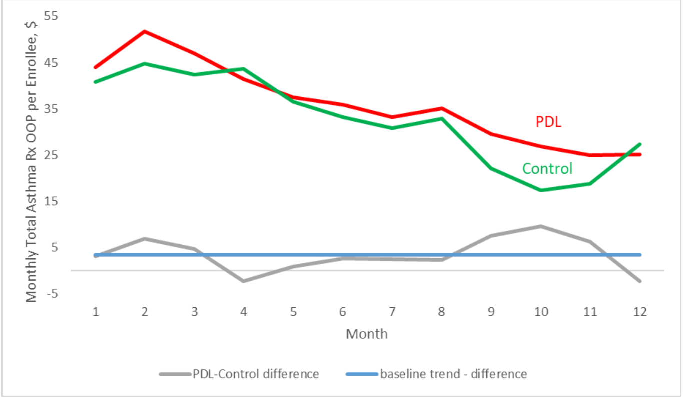

Total asthma OOP costs

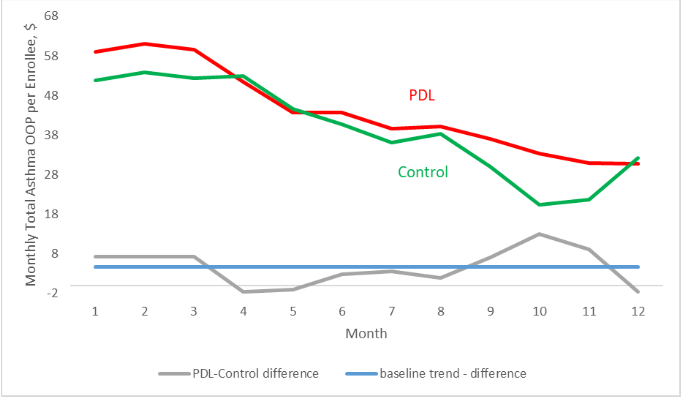

Total OOP costs

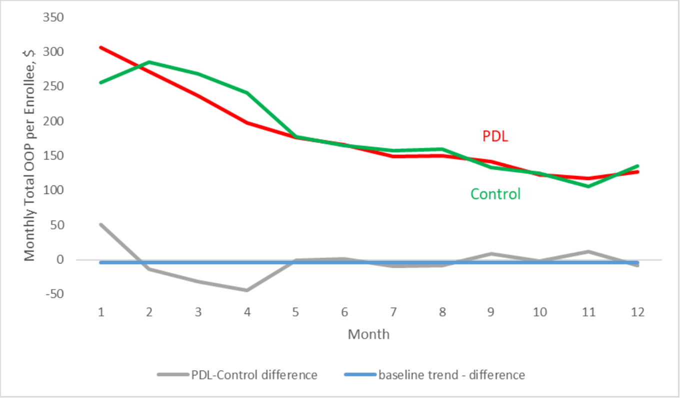

Abbreviations: OOP = out-of-pocket; PDL=preventive drug list

© 2023 Sinaiko AD et al. *JAMA Network Open.*

eTable 1. **Subgroup Analyses Results**: Asthma Controller Medication Use, Adherence, and Exacerbations Before and After Adding a PDL to an HSA-HDHP Compared with a Control Group Remaining in an HSA-HDHP without a PDL, Low-income strata of study population

	**PDL**		**Control**		**Absolute change for PDL vs. control group, (95% CI)** [b]
	pre	post	pre	post	
30-day fills, mean rate per enrollee	n=2,339		n=2,339		
ICS	0.13	0.14	0.12	0.12	0.07 (-0.04 to 0.06)
LTI	0.39	0.40	0.35	0.34	0.02 (-0.05 to 0.09)
ICS-LABA	0.20	0.25	0.23	0.22	0.06 (0.02 to 0.11)[b]
Any controller	0.71	0.79	0.70	0.69	0.09 (-0.01 to 0.19)
Albuterol	0.45	0.44	0.48	0.50	-0.04 (-0.12 to 0.04)
Exacerbations, % [a]					
Any OCS bursts	9.2%	8.1%	9.9%	7.9%	0.8% (-2.0 to 3.7)
Any asthma-related ED visits	1.7%	1.3%	2.2%	2.2%	0.4% (-1.6 to 0.8)

[a] Among those with any controller fill in the baseline period
[b] Statistically significant after Holm-Bonferroni correction.
All n are for the weighted and matched sample.
Low-income strata includes patients who lived in low-income neighborhoods (≥10% of residents below poverty).
Abbreviations: ED = emergency department; ICS = inhaled corticosteroid; ICS-LABA = inhaled corticosteroid-long-acting beta agonist; LTI = leukotriene inhibitor; OCS = oral corticosteroid; PDL = preventive drug list

© 2023 Sinaiko AD et al. *JAMA Network Open.*

eTable 2. **Subgroup Analyses Results**: Asthma Controller Medication Use, Adherence, and Exacerbations Before and After Adding a PDL to an HSA-HDHP Compared with a Control Group Remaining in an HSA-HDHP without a PDL, High-income strata of study population

	**PDL**		**Control**		**Absolute change for PDL vs. control group, (95% CI)** [b]
	pre	post	pre	post	
30-day fills, mean rate per enrollee	n=3,749		n=3,749		
ICS	0.11	0.12	0.08	0.13	-0.05 (-0.11 to 0.01)
LTI	0.35	0.41	0.38	0.40	0.04 (-0.02 to 0.10)
ICS-LABA	0.22	0.26	0.26	0.24	0.06 (0.01 to 0.11)[b]
Any controller	0.67	0.79	0.72	0.77	0.07 (-0.03 to 0.17)
Albuterol	0.51	0.54	0.61	0.57	0.05 (-0.03 to 0.14)
Exacerbations, % [a]					
Any OCS bursts	12.5%	10.7%	12.2%	10.5%	0.0% (-4.4 to 4.3)
Any asthma-related ED visits	3.0%	2.8%	2.9%	2.2%	0.5% (-0.8 to 1.8)

[a] Among those with any controller fill in the baseline period
[b] Statistically significant after Holm-Bonferroni correction.
All N are for the weighted and matched sample.
High-income strata includes patients who lived in neighborhoods with <10% of residents below poverty.
Abbreviations: ED = emergency department; ICS = inhaled corticosteroid; ICS-LABA = inhaled corticosteroid-long-acting beta agonist; LTI = leukotriene inhibitor; OCS = oral corticosteroid; PDL = preventive drug list

© 2023 Sinaiko AD et al. *JAMA Network Open.*

eTable 3. **Subgroup Analyses**: Adjusted Mean Out-of-Pocket Costs Before and After Adding a PDL to an HSA-HDHP Compared with a Control Group Remaining in an HSA-HDHP without a PDL

	**PDL**		**Control**		**Absolute change for PDL vs. control group, (95% CI)**
	pre	post	pre	post	
**Low-income subgroup**	n=2,339		n=2,339		
OOP costs for controller medications					
ICS	$13	$8	$12	$13	-$4 (-$14 to $2)
LTI	$19	$8	$9	$7	-$6 (-$11 to -$1)[a]
ICS-LABA	$30	$19	$27	$24	-$7 (-$15 to $0.2)
OOP costs for albuterol inhalers	$19	$12	$17	$22	-$12 (-$18 to -$7)[a]
OOP costs for all asthma medications	$85	$52	$70	$72	-$36 (-$52 to -$20)[a]
OOP costs for all asthma care	$122	$76	$102	$102	-$46 (-$73 to -$18)[a]
Total OOP costs	$1559	$1481	$1409	$1536	-$219 (-$390 to $48)
**High-income subgroup**	n=3,749		n=3,749		
OOP costs for controller medications					
ICS	$11	$7	$10	$13	-$6 (-$13 to $1)
LTI	$16	$8	$16	$11	-$3 (-$7 to $0.5)
ICS-LABA	$30	$21	$31	$29	-$6 (-$14 to $2)
OOP costs for albuterol inhalers	$21	$15	$26	$28	-$7 (-$11 to -$3) [a]
OOP costs for all asthma medications	$84	$56	$88	$85	-$25 (-$37 to -$13)[a]
OOP costs for all asthma care	$117	$81	$121	$118	-$32 (-$49 to -$16)[a]
Total OOP costs	$1518	$1540	$1486	$1550	-$43 (-$184 to $98)

[a] Statistically significant after Holm-Bonferroni correction
Sample sizes are matched and weighted.
Abbreviations: HDHP = high-deductible health plan; ICS = inhaled corticosteroid; ICS-LABA = inhaled corticosteroid-long-acting beta agonist; LTI = leukotriene inhibitor; OOP = out-of-pocket

© 2023 Sinaiko AD et al. *JAMA Network Open.*
