# Supplementary material for: Utilization and Spending With Preventive Drug Lists for Asthma Medications in High-Deductible Health Plans
Source: JAMA Netw Open. 2023 Aug 29;6(8):e2331259. doi: 10.1001/jamanetworkopen.2023.31259 (PMC10466161; doi:10.1001/jamanetworkopen.2023.31259)
Supplement: Supplement 2. — Data Sharing Statement [file jamanetwopen-e2331259-s002.pdf]

Data Sharing Statement

Sinaiko. Utilization and Spending With Preventive Drug Lists for Asthma Medications in High-Deductible Health Plans. *JAMA Netw Open*. Published August 29, 2023. doi:10.1001/jamanetworkopen.2023.31259

Data
Data available: No

Additional Information
Explanation for why data not available: The study data are subject to Data Use Agreements with the data owner which prohibit our sharing of the data.
